# Circulating Ribonucleic Acids and Metabolic Stress Parameters May Reflect Progression of Autoimmune or Inflammatory Conditions in Juvenile Type 1 Diabetes

**DOI:** 10.1100/tsw.2011.133

**Published:** 2011-07-28

**Authors:** Gordana Kocic, Radmila Pavlovic, Stevo Najman, Goran Nikolic, Dusan Sokolovic, Tatjana Jevtovic-Stoimenov, Dijana Musovic, Andrej Veljkovic, Radivoj Kocic, Natasa Djindjic

**Affiliations:** ^1^ Department of Biochemistry, Medical Faculty University Nis, Serbia; ^2^ Department of Chemistry, Medical Faculty University Nis, Serbia; ^3^ Department of Medical Genetics, Medical Faculty University Nis, Serbia; ^4^ Department of Pathophysiology, Medical Faculty University Nis, Serbia; ^5^ Clinic for Endocrinology, Medical Faculty University Nis, Serbia; ^6^ Medical Faculty University Nis, Serbia

**Keywords:** circulating RNA, peripheral blood mononuclear cells (PBMC), juvenile type 1 diabetes, NF-κB, IRF-3, oxidative stress

## Abstract

The sensing of ribonucleic acids (RNAs) by the monocyte/macrophage system occurs through the TLR7/8 Toll-like receptor family, the retinoic acidi–nducible protein I (RIG-I), and the melanoma differentiation–associated protein-5 (MDA-5). The aim of the present study was to evaluate the effect of circulating RNAs, isolated from juvenile type 1 diabetic patients and healthy control children, on the inflammatory, apoptotic, and antiviral response in human peripheral blood mononuclear cells (PBMCs) isolated from a healthy donor. Obtained effects were compared to the effects of metabolic stress parameters (hyperglycemia, oxidative and nitrosative stress). Forty-eight patients with juvenile type 1 diabetes and control children were included in the study. By performing the chromatographic analysis of circulating RNAs, the peak at the retention time 0.645 min for diabetic and control RNA samples was identified. To determine whether circulating RNAs have an agonistic or antagonistic effect on the signaling pathways involved in inflammatory, apoptotic, and antiviral cascade, their effect on TLR8, RIG-I, MDA-5, MyD88, NF-κB, IRF-3, phosphoIRF-3, IRF-7, RIP, and p38 was evaluated. A significantly lower level was achieved by cultivating PBMCs with circulating RNAs isolated from type 1 diabetic children, compared to the intact PBMCs, in relation to TLR-8, MDA-5, NF-κB, phospho IRF-3, and RIP, while it was higher for Bax. All the metabolic stress conditions up-regulated NF-κB, Bcl-2, and Bax. The NF-κB, determination seems to be the most sensitive parameter that may reflect disease processes associated with the progression of autoimmune or inflammatory conditions, while the IRF3/phosphoIRF3 ratio may suggest an insufficient antiviral response.

## INTRODUCTION

The involvement of the monocyte/macrophage system in the development of type 1 diabetes was emphasized by the lower percentage of the expression of adhesion molecules, impaired mitogenic capacity, and cytokine response[1,2]. The discovery of circulating ribonucleic acids (RNAs) in plasma represents the new diagnostic tool, up to now, for noninvasive prenatal diagnosis or tumor origin diagnosis[3]. Recent experimental evidences have suggested that circulating RNAs would be the epigenetic regulatory molecules involved in cell signaling. The delivery of different RNA species between mammalian cells has been evidenced as ‘‘exosomal shuttle RNA” (esRNA)[4,5,6]. This type of cell communication was documented in tissue development, regeneration, survival, proliferation, cancerogenesis, and in adaptive immune response[4,7]. The different-sized double-stranded RNAs (dsRNAs) or single-stranded RNAs (ssRNAs) may represent the specific determinants for the activation of the innate immune system whenever they are from exogenic origin (known as the pathogen-associated molecular-patterns [PAMP]) or endogenously generated (DAMP)[8]. Introduction of dsRNA fragments into the cytoplasm may change cell antigen presentation (APC) and may induce experimental diabetes[9,10,11]. The three members of the Toll-like receptor family (TLR3, TLR7/8, and TLR9) are located in the endosomal compartment of cells, while the RNA-binding helicases, the retinoic acid–inducible protein I (RIG-I), and the melanoma differentiation–associated protein-5 (MDA-5), are located in the cytosolic compartment[12,13,14]. Stimulation of the TLR pathway activates the myeloid differentiation primary-response protein (MyD88) involved in the early phase of nuclear factor-κB (NF-κB) activation, which leads to the production of inflammatory cytokines[12,15]. The helicase proteins, MDA-5 and RIG-I, sense cytoplasmic RNAs generated during viral replication and activate signals via the interferon promoter stimulator 1 (IPS-1), the latent cytoplasmic transcription factor IFN regulatory factors (IRFs), and the NF-κB[14,15,16]. Activated IRF3 and IRF7 translocate to the nucleus and trigger transcription of IFN-β and IFN-α genes, followed by synthesis and secretion of IFN-α and IFN-β[17,18,19,20]. Functional MDA-5, IRF-3, as well as signal-induced coordination of NF-κB and IRF-3, is required for the optimally controlled and functional host immune response[19].

Our present research is driven by our previous clinical and experimental results, where it was noted that: (1) the level of circulating RNA species and oligonucleotides is higher in diabetes, especially in the juvenile type[21,22,23]; (2) the activity of extracellular RNases toward the ssRNAs and dsRNAs is significantly decreased in diabetes mellitus, presumably because of their time- and dose-dependent glycation[22]; (3) circulating oligonucleotides exerted a peak of maximal absorbance at 270 nm by the spectroscopic scan analysis, equal to cytosine-rich polynucleotide (polyC), compared to different standards[22]; (4) circulating RNAs, isolated from type 2 adult diabetic patients, are able to induce an inflammatory response in isolated thymocytes via NF-κB activation, yet they decreased the antiviral response[24]. It is very intriguing that the macrophage scavenger receptors are unable to recognize polyC[25]. Based on these results, the aim of the present study was to evaluate the effect of circulating RNAs, isolated from juvenile type 1 diabetic patients and healthy control children, on inflammatory, apoptotic, and antiviral response in human peripheral blood mononuclear cells (PBMCs) isolated from a healthy donor, and to compare the observed effects with the *in vitro* effects of metabolic stress parameters, such as hyperglycemia, oxidative and nitrosative stress.

## PATIENTS AND METHODS

### Patients and Healthy Control Subjects

Forty-eight children with juvenile type 1 diabetes (age group 2.5–14 years) and a control group of healthy children (n = 22, age group 3.5–14 years) were included in the study. All diabetic patients were recruited from the Nis South-East Regional University Hospital, Serbia, for a period of about 1 year (September 2008 to September 2009). All patients were divided into two age groups: preschool age (under 7 years) and school age (7–14 years). The diagnosis of diabetes was made according to criteria of the World Health Organization (WHO)[26]. Beside the classical symptoms of diabetes, the level of C-peptide (<0.20 nmol/l) was used as an indicator of low β-cell function. The metabolic regulation of the disease was monitored according to glycemia and HbA1c levels (Table 1). Anthropometric measurements were performed using standardized techniques. The exclusion criteria from the study were diabetic or nondiabetic renal disease, acute or chronic infection. Patients with other autoimmune diseases, immunodeficiency, or under any immunosuppressive therapy were excluded from the study as well. The mean [±SD] daily dose of insulin was 0.72 ± 0.21 U/kg per body weight per·day for the preschool age group and 0.68 ± 0.28 U/kg per body weight per·day for the school age group. The clinical evaluations were performed on the same day as the blood collection. The control group was comprised of age-matched children, clinically healthy, coming for routine medical check-ups (necessary for applying for admission to a nursery, primary, or secondary school) for the above-mentioned period. The exclusion criteria were any sign of infections, immune disorders, or hyperglycemia (mean glucose level of control group was 3.95 ± 0.38 mmol/l).

**TABLE 1**
**Main Clinical Characteristics of Children with Juvenile Type 1 Diabetes**

| Investigated Parameters | Glycemia (mmol/l) | HbA1c (%) | BMI (kg/m$^2$) | Duration of Disease (Year) | Age |
|---|---|---|---|---|---|
| Juvenile Type 1 diabetes (age under 7) (n = 12) | 10.01 ± 5.01 | 9.22 ± 3.52 | 18.32 ± 3.01 | 1.37 ± 0.68*** | 5.00 ± 1.15*** |
| Juvenile Type 1 diabetes (age 7–14) (n = 36) | 9.02 ± 3.26 | 8.21 ± 2.04 | 18.77 ± 2.91 | 5.35 ± 3.99 | 12.35 ± 2.05 |

*** $p < 0.001$.

### Chemicals

RNA was purchased from Calbiochem (La Jolla, CA, USA). Different monoclonal primary or secondary antibodies were purchased from Santa Cruz Biotechnology (Santa Cruz, CA, USA). All other chemicals and culture medium were purchased from Sigma (St. Louis, MO, USA).

### Preparation of Samples and Experimental Methods

The blood samples for plasma separation of juvenile type 1 diabetic patients and the healthy control children were collected into heparin-containing sterile Vacutainer® tubes (Becton, Dickinson, Franklin Lakes, NJ, USA) and the blood was centrifuged for 10 min at 1800 *g* according to the protocols for RNA isolation. Supernatant was removed and centrifuged for a second time at 1300 *g* for 10 min to eliminate any remaining cells.

### Isolation of RNA and Oligonucleotide Samples

For the isolation of RNA from plasma samples, the commercial RNA isolation reagent set, TRI Reagent® BD (T3809), for the simultaneous isolation of RNA, DNA, and proteins from plasma was used, purchased from Sigma. This test was the most efficient for isolation of short RNA fragments, giving the best recovery results. Plasma RNA isolation was performed according to the manufacturer's instructions and data were compared according to the published results[22,23,24]. Plasma concentrations were calculated by employing the corresponding standard[27]. Purified RNA was employed for culture media, for the spectrophotometric scan analysis, and HPLC analysis. Each plasma sample was done in duplicate. For culture media, the pellet of RNA was dissolved in 1000 µl of PBS; for HPLC chromatographic analysis, the RNA pellet was dissolved in the HPLC mobile phase. The blank sample was prepared in the same way, except that distilled water was used instead of plasma specimen.

### HPLC Analysis of Plasma RNA Samples

The obtained plasma RNA pellet was characterized using a series 1200 Agilent HPLC system consisting of a binary gradient pump, a diode array detector (DAD) (set at 270 nm), an autosampler, a solvent degasser system, and a column thermostat. Chromatographic separation was achieved with a Helix DVB column (50 × 3 mm) (Agilent Technologies, Santa Clara, CA, USA) and the column temperature was set at 50$^{o}$C. Two buffers were used to form a solvent gradient: buffer A containing 95% of 100 mM triethylammonium acetate (TEAA), pH 7.0, and 5% of acetonitrile; and buffer B comprising 85% 100 mM TEAA, pH 7.0, and 15% acetonitrile. Flow rate was 1 ml/min, with an increase of buffer B of 0.15%/min from 0 to 10 min, and maintained at the obtained level up to the end of the run (16 min). The samples were reconstructed in 100 µl of the mobile phase, and 10 µl for the blank and diabetic group was injected, while in the case of the control group, because of low concentration, 30 µl was analyzed.

### *In vitro* Experiments on PBMCs

Peripheral venous blood (350 ml) from a 24-year-old healthy male volunteer was drawn between 0800 and 0900 h into sterile heparinized tubes and was processed within 2 h. PBMCs were isolated under sterile conditions by centrifugation in Ficoll Histopaque® 1077 (Lymphoprep™, Nycomed Pharma, Zurich, Switzerland) according to the manufacturer's instructions. Cell viability was above 90% using Trypan blue stain exclusion. After washing in PBS, obtained PBMCs were resuspended in RPMI 1640 medium and 10% FCS.

For the evaluation of isolated circulating RNA effects, washed PMBCs (each aliquot of 100 µl contained $2 \times 10^5$ cells) were distributed in eight 12-well plates. An aliquot of either 100 µl of RNA solutions (isolated from diabetic and healthy control plasma samples) or PBS alone (untreated group of PBMCs) was added to each well. PBMCs were cultured in a total volume of 1000 µl (100 µl of cells/well in 800 µl of RPMI 1640 medium and 100 µl of RNA) for 4 h at 37°C in 5% $CO_2$. Afterwards, the supernatants were discarded and cells were washed and resuspended in 1000 µl of PBS. The 50-µl aliquots of each sample were plated in 12 U-bottom 96-well culture plates for the determination of TLR-8, MyD88, NF-κB, Bcl-2, Bax, p38, RIG-I, RIP, MDA-5, IRF-3, phosphoIRF-3, and IRF-7.

For the evaluation of metabolic stress parameters, the isolated PBMCs were cultured with medium containing high glucose (10, 50, and 100 mmol/l glucose), hydrogen peroxide ($H_2O_2$) (0.01, 0.1, and 0.5 mmol/l), nitric oxide (NO) (0.1, 1.0, and 10 mmol/l sodium-nitroprusside), and peroxinitrite (ONOO) (0.01, 0.1, and 0.5 mmol/l) solutions for 4 h at 37°C in 5% $CO_2$. Cultures were prepared in 12-well plates using six wells per condition. The synthesis of authentic ONOO was explained in our previous report[28]. At the end of the incubation time, the supernatants were discarded, then cells were washed and resuspended in 1000 µl of PBS. Afterwards, the 50-µl aliquots of each sample were plated in seven U-bottom 96-well culture plates for the determination of NF-κB, Bcl-2, Bax, MDA-5, IRF-3, phosphoIRF-3, and IRF-7.

### Detection of TLR-8, MyD88, NF-κB, Bcl-2, Bax, p38, RIG-I, RIP, MDA-5, IRF-3, phosphoIRF-3, and IRF-7

The PBMCs, resuspended in U-bottom 96-well culture plates, were fixed by using 70% methanol and permeabilized with 0.1% Triton PBS. Specific antibodies used were anti-TLR8 (D-14 sc-17585), anti-

MyD88 (B-1 sc-136970), anti–RIG-I (C-15 sc-48929), anti–MDA-5 (C-16: sc-48031), anti–NF-κB (p65 C-20: sc-372 epitope mapping at the C-terminus of NF-κB p65), anti–IRF-3 (FL-425: sc-9082), anti–phosphoIRF-3 (C-20 sc-15994), anti–IRF-7 (C-20 sc-15994), anti–Bcl-2 (N-19: sc-492), anti-Bax (P-19: sc-526), anti-RIP (C-12 sc-133102), and anti-p38 (p38α/β A-12: sc-7972). Corresponding antimouse, -goat, and -rabbit secondary fluorescein (sc-7972 FITC) antibodies were employed as needed. The cells were incubated with corresponding primary antibody, washed three times, and incubated with the FITC-conjugated secondary antibody. The excess of antibody following staining was washed by adding PBS to each well. The mean fluorescence intensity (MFI; logarithmic scale) of cell populations was determined and analyzed on a Victor™ multiplate reader (Perkin Elmer-Wallace, Wellesley, MA). The results presented were obtained following subtraction of blank values treated with secondary antibodies only. The fluorescence intensity of cells indicated for both up and down regulating populations for each stimulus used, compared to intact PBMCs (treated with PBS instead of RNA or metabolic stress parameters), and calculated as a percent change when control PBMCs were set at 100%.

### Statistical Methods

The results are presented as mean ± SD. The statistical significance was evaluated between each group of PBMCs treated with RNA (isolated from type 1 diabetic children and control group) and intact untreated PBMCs, and between PBMCs treated with RNA of diabetic or healthy control samples and between the groups incubated under metabolic stress conditions and intact PBMCs, by using ANOVA test. The limit significance for all analyses was defined as $p = 0.05$.

## RESULTS

The clinical characteristics of juvenile type 1 diabetic children recruited for the study are shown in Table 1. The concentration of circulating RNAs was significantly higher in diabetic patients compared to control values (Table 2). No significant difference was observed regarding the circulating RNA level in the two age groups of diabetic children. By performing HPLC analysis by using a Helix DVB column, the chromatograms obtained by HPLC-DAD techniques identified the peaks at the retention time 0.645 min for diabetic and control RNA samples (Fig. 1A,B). Blank samples (performed in the same way, except that water was used instead of plasma) did not appear at the mentioned retention time (Fig. 1C). Characteristic UV spectrum of extracted peaks again confirmed maximal absorbance at 270 nm (Fig. 1D), as already obtained by spectrophotometric scan analysis[22].

**TABLE 2**
**Plasma RNA Concentration of Investigated Groups**

| Investigated Parameter | Juvenile Type 1 Diabetes (age under 7) (n = 12) | Juvenile Type 1 Diabetes (age 7–14) (n = 36) | Healthy Control Children (n = 22) |
|---|---|---|---|
| Plasma RNA concentration (mg/l) | 137.22 ± 17.82*** | 149.20 ± 37.12*** | 75.98 ± 11.77 |

*Note*: For the isolation of RNA from plasma samples, the commercial RNA isolation reagent set, TRI Reagent® BD (T3809), for the simultaneous isolation of RNA, DNA, and proteins from plasma was used. The RNA isolation was performed according to the manufacturer’s instructions. Plasma concentration was calculated by using corresponding standard.

*** $p < 0.001$ (compared to the control value).

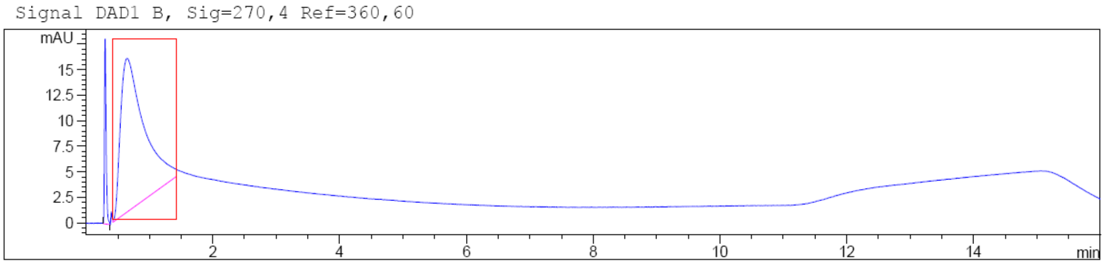

**A**

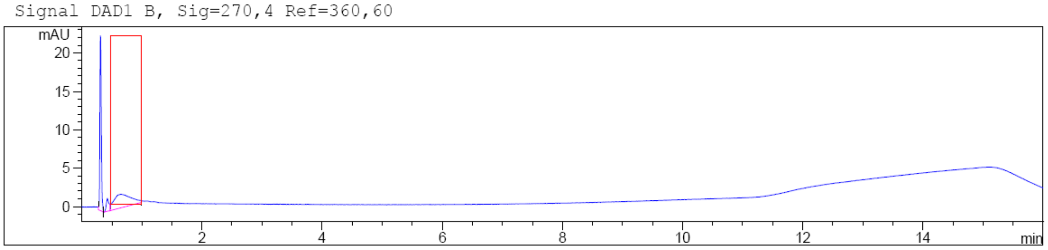

**B**

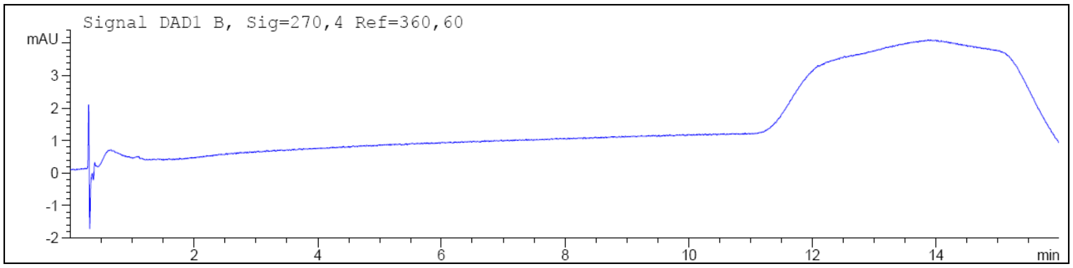

**C**

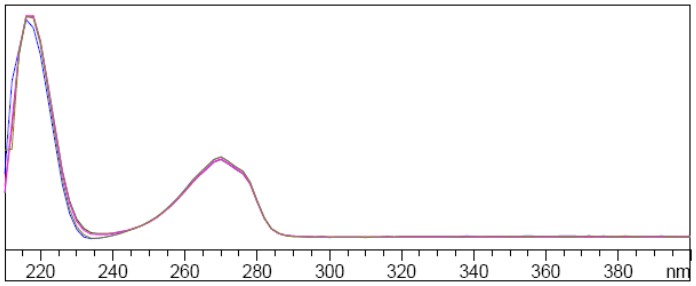

**D**

**FIGURE 1.** HPLC-DAD chromatograms with peak at retention time 0.645 min: (A) sample from children with type 1 diabetes, (B) controls, (C) blank probe, (D) characteristic UV spectrum of extracted peak. Circulating RNA oligonucleotides were characterized using a series 1200 Agilent HPLC chromatographic separation achieved with a Helix DVB column (50 × 3mm) and the column temperature was set at 50°C. Flow rate was 1 ml/min with an increase of buffer B of 0.15%/min from 0 to 10 min, and maintained at the obtained level up to the end of the run (16 min). The samples were reconstructed in 100 µl of the mobile phase, and 10 µl for the blank and diabetic group was injected, while in the case of the control group, 30 µl was analyzed.

To determine whether circulating RNAs have an impact on the signaling pathways involved in inflammatory, apoptotic, and antiviral cascade, a high-throughput method was used to determine whether RNAs may be recognized by TLR8, RIG-I, or MDA-5, and whether they act either in an agonistic or antagonistic manner. The method is based on the modulation of various transcription factors, including MyD88, NF-κB, IRF-3, phosphoIRF-3, IRF-7, RIP, and p38. We also determined the possible apoptotic cellular effect by evaluating the Bcl-2 and Bax response. A significantly lower concentration of TLR-8, MDA-5, NF-κB, RIP, RIG-I, IRF-3, phosphoIRF-3, and IRF7 (determined by the fluorescence intensity) was achieved by cultivating PBMCs with RNAs isolated from children with type 1 diabetes, compared to the intact PBMCs (Figs. 2–4). Concerning the effects on NF-κB, obtained results are opposite to our previously published results related to adult type 2 diabetes or adult patients with the end-stage renal failure[23,29]. Since the transcription factor complex required for IFN gene induction is composed of phosphoIRF3 dimer and/or phosphoIRF-3/IRF7, of importance was the relationship between the inactive IRF-3 and the phosphorylated IRF-3 in different groups of cells. In PBMCs treated with control RNA samples, the IRF3/phosphoIRF3 ratio was <1, while in PBMCs treated with the RNAs isolated from juvenile type 1 diabetic patients, it was >1 in more than 80% of the samples. In resting, untreated cells, the ratio was near 1 (Fig. 5). Circulating RNAs significantly up-regulated Bax (Fig. 3). Only mild Bcl-2 and MyD88 up-regulation was obtained (Figs. 2 and 3). The Bcl-2/Bax ratio was lower in PBMC samples when treated with circulating RNAs from diabetic patients, which suggests that the circulating RNAs may provoke a proapoptotic response. No significant response was obtained for p38 (Fig. 4).

Metabolic dysregulation of diabetes may induce inflammatory cascade[30,31]. In order to test this observation *in vitro*, the PBMCs were exposed to high glucose, $H_2O_2$, NO, and ONOO. The results are shown in Table 3. The cellular levels of NF-κB, MDA-5, Bcl-2, Bax, IRF-3, phosphoIRF-3, and IRF-7 were determined. All the metabolic stress conditions were able to elicit a strong NF-κB, Bcl-2, and Bax up-regulation. The highest NO and ONOO concentration elicited a weaker response. All the metabolic stress parameters down-regulated MDA-5, IRF-3, phosphoIRF-3, and IRF-7.

### DISCUSSION

During the development of type 1 diabetes, the immune system recruits monocyte-derived cell populations to orchestrate the innate immunity response[31,32]. The response is determined by complex interaction between possible pathogens (PAMP molecules), tissue injury molecules (DAMP molecules), oxidatively modified molecules, or metabolic stress parameters (hyperglycemia, oxidative or nitrosative stress), resulting in expression of a variety of inflammatory cytokines or induction of the tissue repair mechanisms[33,34,35,36]. The sensing of the RNAs by immune cells occurs via specific immunoreceptors, according to the preferences for structural motifs or according to intracellular compartmentation[12,13,14,15,16]. The extracellular nucleases degrade circulating nucleic acids and prevent ligand-induced stimulation of immune and inflammatory responses[37]. Several reports have documented the presence of circulating extracellular RNAs in serum, protected from degradation by plasma ribonucleases (RNase)[37]. One hypothesis would be that the RNAs are protected via binding to the lipoprotein complexes or sequestered within lipid vesicles. After the packaging into vesicles, the RNAs may be fused with the target cell. Afterwards, the released RNAs may mediate epigenetic modifications[6,7]. In our previous report, we also documented significantly decreased RNase activity in diabetic patients, presumably due to the time- and dose-dependent enzyme glycation[22]. In monocytes, the response to RNAs is presumably mediated by either TLR8 or RIG-I[14,38]. A number of viral infections are associated with the production of RNA species in the absence of infection, such as the different genomic fragments, replicative intermediates, and stem-and-loop structures[8]. TLR interactions with the "self" molecules, such as self-DNA, self-RNA, and the self-nucleotide complexes, represent the initial trigger for development of autoimmune diseases, known as the toll hypothesis, first explained in systemic lupus erythematosus[31]. In our study, RNAs from plasma specimens were significantly higher in juvenile type 1 diabetic patients (Table 2). Incubation of PBMCs with the circulating RNAs from juvenile

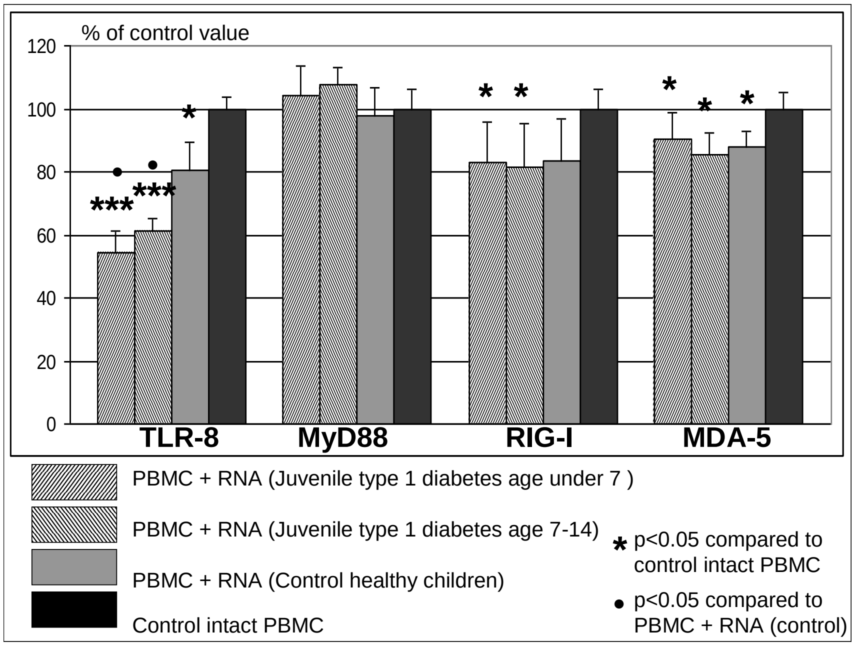

**FIGURE 2.** Quantitation of TLR8, MyD88, RIG-I, and MDA-5 in PBMCs exposed to circulating RNAs. For the evaluation of isolated circulating RNA effects, washed PMBCs were distributed in 12-well plates. The RNA solutions (isolated from diabetic and healthy control plasma samples) or PBS alone (untreated group of PBMCs) were added to each well. PBMCs were cultured in a total volume of 1000 µl for 6 h at 37°C in 5% $CO_2$. Afterwards, the cells were washed and the 50-µl aliquots of each sample were plated in U-bottom 96-well culture plates for the determination of TLR8, MyD88, RIG-I, and MDA-5 by using 70% methanol and permeabilized with 0.1% Triton PBS. Specific antibodies were employed. The mean fluorescence intensity (MFI; logarithmic scale) of cell populations was determined and analyzed on a Victor™ multiplate reader, and calculated as a percent change when control PBMCs were set at 100%.

type 1 diabetic patients decreased immunofluorescence by using specific anti-TLR8, anti–MDA-5, anti–RIG-I, and anti–NF-κB antibodies, which may contribute to their antagonistic effect (Fig. 2). The activation of TLR8 and the helicase systems RIG-I and MDA-5 up-regulate NF-κB and induce consequent production of the proinflammatory cytokines. In resting cells, NF-κB is inactive as a result of its association with the IκB family of inhibitory proteins, existing in the cytoplasm as a heterodimer of p50/p65 subunits[39]. Transcriptional factor NF-κB has a potent antiapoptotic function, acting as a transcriptional switch of the inflammatory response via the regulation of gene expression for cytokine production, cell adhesion, lymphocyte maturation, antigen processing, and presentation by MHC class I molecules. In models of experimental autoimmune diabetes, the NOD mouse and diabetes-prone BB (BBDP) rat, TLR up-regulation can suppress the onset of the disease, which is consistent with our findings. In the same model, the lymphoid subpopulations exert increased susceptibility to apoptosis and impaired activation of the transcription factor NF-κB, as a result of mutation in the promoter-enhancer region of the gene for the proteasome subunit[40]. The inhibition of the NF-κB signaling was documented as the main target for viruses to escape immune defenses[39]. The specter of NF-κB gene alterations varies in different autoimmune diseases, but usually decreased NF-κB activity, in response to selected cytokines, was noted[41,42]. In our previous reports, the circulating RNAs isolated from adult patients with type 2 diabetes and from adult patients with end-stage renal failure induced an inflammatory response

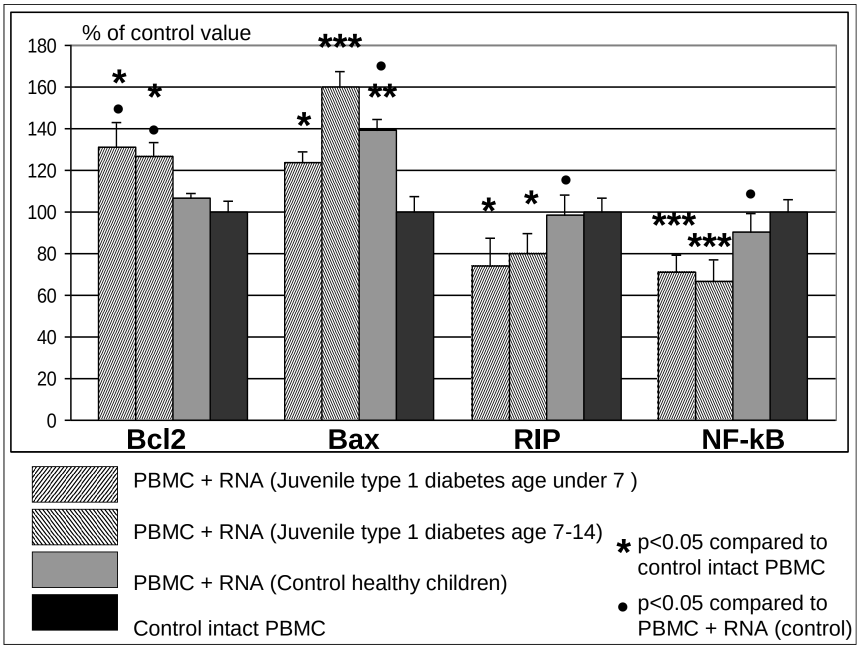

**FIGURE 3.** Quantitation of Bcl-2, Bax, RIP, and NF-κB in PBMCs exposed to circulating RNAs. Evaluation performed as described for Fig. 2.

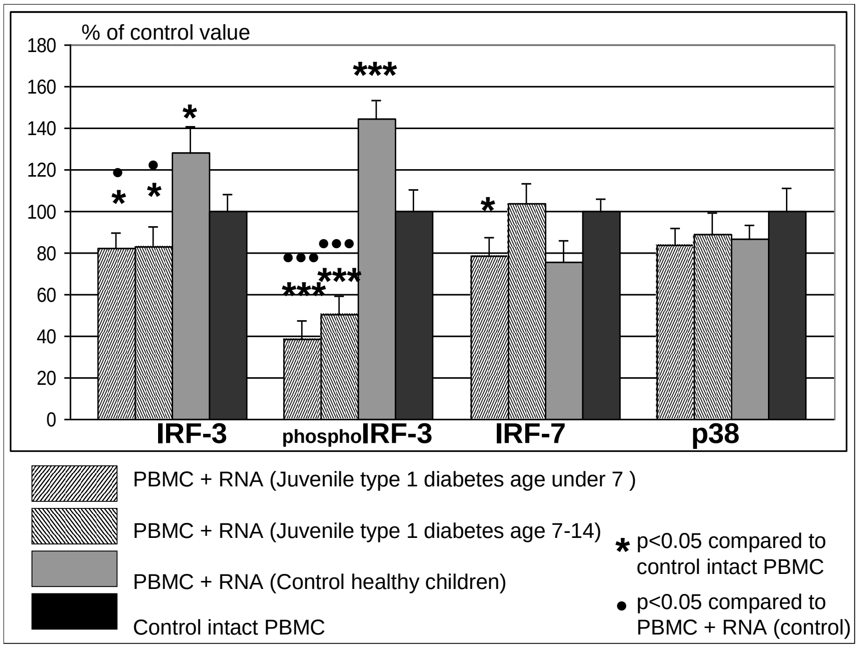

**FIGURE 4.** Quantitation of IRF-3, phosphoIRF-3, IRF-7, and p38 in PBMCs exposed to circulating RNAs. Evaluation performed as described for Fig. 2.

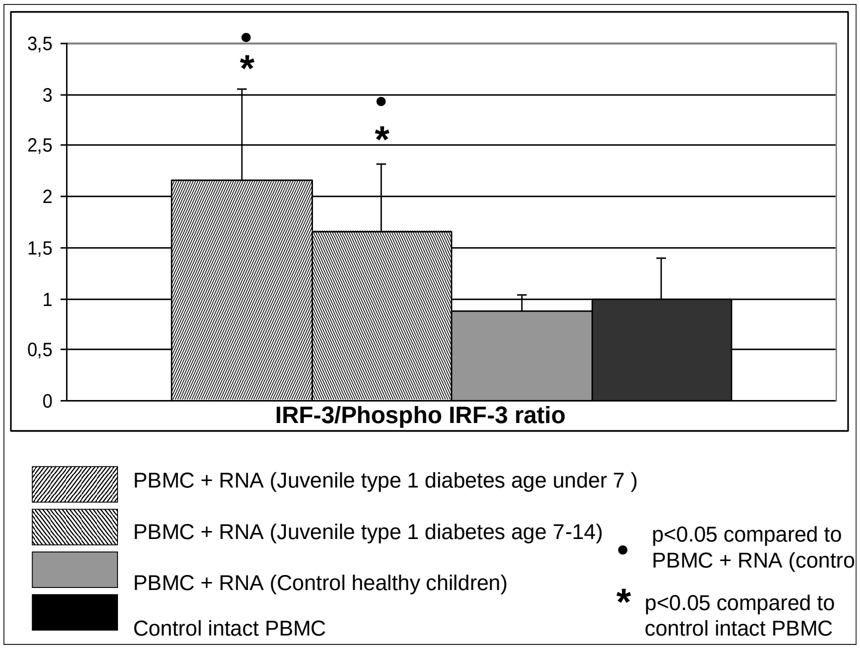

**FIGURE 5.** Quantitation of the IRF-3/phosphoIRF-3 ratio in PBMCs.

through NF-κB activation[24,29]. Susceptibility to viral infections or to specific bacterial strains is higher in diabetic patients compared with nondiabetic individuals[43,44]. The importance of IRF-3 in antiviral response was documented by using IRF-3$^{-/-}$ mice, which are more vulnerable to viral infection. IRF-3 cooperates with NF-κB and activates transcription factor-2/c-Jun in order to make a transcriptionally active enhanceosome complex. Different kinases can phosphorylate serine residues of IRF-3 and thereby activate it. Afterwards, the IRF-3 homo- or heterodimerizes with IRF-7, translocates to the nucleus, and associates with the coactivator. Both IRF3 and IRF7 were documented to be activated by the same mechanism, and showed the same sensitivity and resistance. The phosphorylation mechanism contributes to the ability of IRF-3 to homodimerize or associate with IRF-7, and regulate its transcriptional activity[19,20]. In our study, the inverse ratio of phopshoIRF-3 to intact IRF-3 was observed after the PBMCs were treated with the RNAs of juvenile diabetes type 1, while in the cells treated with the RNA samples from the control children, the ratio was <1. Obtained data may suggest that some RNA bases may alter phosphorylation reactions and the IRF signaling, which may have an influence on further antiviral response. The observed RIP inhibition may also contribute to NF-κB down-regulation (Fig. 3), while the level of p38 exerted no significant response (Fig. 4).

We explored the effect of metabolic stress parameters, in an effort to define the major contributors for the inflammatory cascade in diabetes type 1. Results of our *in vitro* study documented that the metabolic stress parameters (hyperglycemia, oxidative and nitrosative stress) up-regulated NF-κB. The NF-κB is documented as a typical "redox-sensitive" transcription factor, where the reactive oxygen species (ROS) induce the phosphorylation of the IκB subunit. It was documented that the high glucose conditions may induce an inflammatory state, accompanied with the deficient immune cellular response. Studies about increased NO secretion, due to the local induction of "inducible" NOS-2, suggested that NO may have an impact on development and progression of atherosclerosis and diabetic complications[45,46,47,48]. The development of macrovascular complications is usually seen in older diabetic patients[30]. Obtained results may indicate that the magnitude of NF-κB modulation may (re)direct for inflammatory cascade in type 1 diabetes, precipitating in mechanisms of development of diabetic atherosclerotic complications.

**TABLE 3**
**Effect of Metabolic Stress Parameters on NF-κB, MDA-5, Bcl-2, Bax, IRF-3, phosphoIRF-3, and IRF-7**

| Investigated Parameters (% of control PBMCs) | Glucose (10, 50, 100 mmol/l) | $H_2O_2$ (0.01, 0.1, 0.5 mmol/l) | NO (0.1, 1.0, 10 mmol/l) | ONOO (0.01, 0.1, 0.5 mmol/l) |
|---|---|---|---|---|
| NF-κB | 110.0 ± 6.21 | 130.99 ± 8.94* | 130.0 ± 15.09* | 133.97 ± 12.48* |
| | 210.0 ± 14.53*** | 211.0 ± 14.34*** | 150.0 ± 7.57** | 192.98 ± 12.98*** |
| | 262.42 ± 24.9*** | 323.54 ± 21.67*** | 113.35 ± 12.36* | 182.98 ± 14.59*** |
| MDA-5 | 92.99 ± 4.11 | 82.05 ± 3.17* | 75.98 ± 9.34* | 80.21 ± 3.83* |
| | 75.88 ± 3.83* | 72.05 ± 11.40* | 80.01 ± 11.4 | 75.14 ± 4.76* |
| | 67.75 ± 4.67* | 80.0 ± 10.18* | 75.09 ± 5.14* | 74.11 ± 3.08** |
| Bcl-2 | 123.3 ± 14.47* | 101.38 ± 10.83 | 135.18 ± 16.20** | 111.0 ± 8.57 |
| | 132.49 ± 7.71** | 121.66 ± 12.39* | 175.73 ± 5.80*** | 107.36 ± 9.27 |
| | 96.79 ± 7.53 | 242.98 ± 12.91** | 120.71 ± 11.61 | 99.91 ± 9.90 |
| Bax | 130.04 ± 9.44* | 105.93 ± 6.17 | 113.99 ± 4.05* | 104.54 ± 6.23 |
| | 121.62 ± 5.33* | 111.50 ± 4.05** | 115.99 ± 5.39** | 117.02 ± 10.05* |
| | 122.53 ± 5.39 | 197.03 ± 5.33*** | 125.01 ± 5.45*** | 145.12 ± 6.17* |
| IRF-3 | 98.76 ± 7.88 | 87.93 ± 3.69* | 88.91 ± 7.63** | 82.02 ± 4.67* |
| | 70.93 ± 4.67** | 82.01 ± 17.24** | 85.96 ± 4.43** | 80.29 ± 7.14* |
| | 85.96 ± 17.98* | 86.94 ± 5.42*** | 75.86 ± 7.88*** | 74.87 ± 7.63* |
| PhosphoIRF-3 | 100.75 ± 5.82 | 103.79 ± 10.37 | 77.72 ± 15.94 | 81.26 ± 6.58* |
| | 92.65 ± 11.89 | 98.48 ± 6.83 | 73.16 ± 4.81** | 75.44 ± 9.62** |
| | 76.45 ± 8.60** | 88.60 ± 3.03* | 67.34 ± 4.05*** | 69.87 ± 7.59*** |
| IRF-7 | 88.47 ± 2.40 | 95.91 ± 3.44 | 89.99 ± 3.20* | 88.47 ± 2.32* |
| | 82.14 ± 2.80 | 81.34 ± 2.64* | 85.98 ± 10.80* | 74.29 ± 9.60* |
| | 79.50 ± 3.20 | 80.30 ± 10.16* | 77.98 ± 2.32*** | 74.37 ± 10.56* |

*Note:* For the evaluation of metabolic stress parameters, the isolated PBMCs were cultured with medium containing high glucose (10, 50, and 100 mmol/l glucose), $H_2O_2$ (0.01, 0.1, and 0.5 mmol/l), NO (0.1, 1.0, and 10 mmol/l sodium-nitroprusside), and ONOO (0.01, 0.1, 0.5 mmol/l) solutions for 4 h at 37°C in 5% $CO_2$. The synthesis of authentic ONOO was explained in our previous report. The 50-µl aliquots of each sample were plated in U-bottom 96-well culture plates for the determination of NF-κB, Bcl-2, Bax, MDA-5, IRF-3, phosphoIRF-3, and IRF-7. The mean fluorescence intensity (MFI; logarithmic scale) of cell populations was determined on a Victor™ multiplate reader and calculated as a percent change, when control PBMCs were set at 100%.

* $p < 0.05$; ** $p < 0.001$; *** $p < 0.001$ (compared to control PBMCs).

In response to different death signals, the oligomerization of proapoptotic proteins Bax and Bak induces release of proapoptotic factors, such as cytochrome *c* to the cytosol that activates the effector caspase-3 and endonucleases[49]. The antiapoptotic protein Bcl-2 prevents the Bax/Bak oligomerization. In our study, Bax up-regulation was observed after PBMC incubation with circulating RNAs from type 1 juvenile diabetic patients (Fig. 3), as well as upon stimulation in metabolic stress conditions (Table 3). Based on the estimated Bcl-2/Bax ratio, they may be in a proapoptotic condition. Our results are in agreement with the significant inhibition of the proliferative responses to mitogens and the respiratory burst activity of PBMCs seen in diabetic patients[2].

The ability of the investigated metabolic stress conditions to down-regulate MDA-5, IRF-3, phosphoIRF-3, and IRF-7 may have an influence on affected antiviral responses. Inadequate metabolic control decreases PBMC immune response and may be a possible cause of the increased incidence of infections in type 1 diabetic patients[1,43,44].

In conclusion, our observation is that circulating RNAs may modulate the inflammatory and apoptotic pathway, which may lead to impaired cellular antiviral responsiveness in juvenile diabetes type 1. NF-κB may function as the cellular checkpoint of metabolic stress conditions, such as hyperglycemia, oxidative and nitrosative stress, which may reflect disease processes associated with progression of autoimmune or inflammatory conditions.
